# The effect of familiarity and dog’s body size on female owners’ dog-directed communication

**DOI:** 10.1007/s10071-025-02041-1

**Published:** 2026-01-08

**Authors:** Lőrinc András Filep, Édua Koós-Hutás, Fanni Hollay, József Topál, Anna Gergely

**Affiliations:** 1 HUN-REN Hungarian Research Center for Natural Sciences, Institute of Cognitive Sciences and Psychology, Budapest, Hungary; 2 Doctorate School of Psychology, Eötvös Loránd University, Budapest, Hungary; 3 NAP 3.0 Comparative Ethology Research Group, Budapes, Hungary

**Keywords:** Prosody, Familiarity, Dog, Human-dog interaction, Facial expression

## Abstract

Exaggerated prosody directed toward dogs has multiple functions, including attention getting and maintaining as well as expressing positive emotions toward the canine partner. However, the role of prosody in owner-dog familiarity remains unclear. To address this gap, we examined the effect of familiarity on the acoustic and visual prosodic features of dog-directed speech. To this end, we analyzed the prosodic features of female speakers when interacting with their own dogs vs. an unfamiliar dog of the same breed during three different situations: *Attention-getting*, *Task-solving*, and *Nursery rhymes*. Interestingly, only the mean pitch was affected by familiarity as speakers used a higher pitch when interacting with the unfamiliar dog compared to their own dog. Meanwhile, pitch range and the intensity of ‘happy’ facial expressions were affected by the dog’s size: speakers used a wider pitch range and more intense ‘happy’ expressions when interacting with smaller dogs. The *Nursery rhymes* situation evoked the most intense visual prosody, perhaps due to the use of nursery rhymes when interacting with infants. Our results suggest that a heightened overall pitch may serve as a way to appear friendly toward an unfamiliar being, acting as a universal engaging mechanism in human-dog communication. In contrast, the ‘happy’ facial expression and pitch range seem to be less sensitive to the familiarity with a dog, being more influenced by the specific situation and size of the canine partner which is likely related to the ‘cuteness factor’ and/or the baby schema in female speakers.

**Supplementary Information:**

The online version contains supplementary material available at 10.1007/s10071-025-02041-1.

## Introduction

Dog-directed speech is a relatively new yet growing area of research, the relevance of which is twofold. First, due to the increasing global popularity of companion dogs, understanding the nuances of human-dog interaction has become more valuable (e.g. Paxton 2000). Second, it has been proposed that human-dog communication may offer a basis for studying the functions of prosodic features (e.g. Gergely et al. 2017; Hirsh-Pasek and Treiman 1982). These are generally grouped into two categories, acoustic prosody (e.g. fundamental frequency / *f0* or pitch range) and visual or facial prosody (i.e. facial movements that accompany certain prosodic speech and form facial expressions to convey emotional states, Chong et al. 2003; Gergely et al. 2023a, b). Recent studies comparing dog-, infant- and adult-directed communication styles have provided significant insights into the acoustic and facial prosodic features and their roles toward partners who differ in their cognitive capabilities, emotional needs, and the nature of their relationship with the speaker (Gergely et al. 2017, 2023a, b; Koós-Hutás et al. 2024, 2025).

Infant-directed speech (or ‘motherese’) has a longer research history and its functions are relatively well understood (for a review see Soderstrom 2007). The focus of the present paper is to investigate a lesser-studied factor that may influence acoustic and / or visual prosodic elements: the familiarity between interactants (see for example Bowlby, 1982 and Fernald 1992 for accounts on attachment). Research evidence suggests that pet dogs tend to form attachments with humans that are similar to those infants form with their primary caregivers (e.g. Prato-Previde et al. 2003; Topál et al. 1998), so it seems logical that adult humans use comparable prosodic strategies with dogs and infants with whom they are in a close relationship. Previous studies have provided important insights into the effects of familiarity / bonding on prosody directed toward infants, adults and dogs. Motherese, parentese or baby-talk is listed amongst the so-called ‘attachment vocalizations’ of humans that are thought to have evolved to increase the probability of survival by strengthening bonds between caregivers and their relatively undeveloped infants (e.g. Chang 2013; Falk, 2004). Besides ‘motherese’, there is growing evidence from adult-adult interactions that emotional attachment has a great influence on prosody. For instance, baby-talk-like acoustic prosody can occur between adults in romantic relationships which is often referred to as lover’s talk (e.g. Bombar & Littig 1996). Moreover, visual prosody in spouse-directed speech resembles infant-directed prosody as both are marked by more intense and frequent ‘happy’ facial expressions – features that are rare or completely missing from interactions with friendly but unfamiliar adults in similar situations (Gergely et al. 2023a, b; Koós-Hutás et al. 2025). (‘Happy’ facial expression here stands for a pre-made feature in Noldus Face Reader that identifies a smile with the cheeks raised as well, i.e. Duchenne-smile.) Last but not least, familiarity was also identified as an influential factor in dog-directed speech (Mitchell 2004). In this study, both female and male speakers’ play sessions with their own dog and an unfamiliar dog were recorded in a within-subject design. The lexical content, complexity and repetitiveness of their dog-directed speech were analyzed in relation to familiarity. However, prosody was assessed as a binary variable: based on at least two of three coders’ agreement, each unit of speech was categorized as either baby-talk or not (Mitchell 2004). When interacting with unfamiliar dogs, speakers tended to have more of a ‘conversation’, i.e. they used longer utterances, more varied vocabulary, and attempted ‘to appear friendly’. Female speakers generally used more baby-talk with dogs, but both sexes used baby-talk more frequently when playing with an unfamiliar dog than with their own dog (Mitchell 2004). This finding might seem surprising from the point of view of human-human interactions, as in this case, the presence of the emotional bond was found to predict more intense prosody (Gergely et al. 2023a, b; Koós-Hutás et al. 2025), whereas with dogs, the opposite seems to be true, i.e. it is the lack of familiarity that elicits intense prosodic features. In another study, Lesch and co-workers (2019) recorded and analyzed the acoustic parameters of owners’ voices directed at their dogs during the well-known Ainsworth Strange-situations test, a standardly used paradigm for assessing attachment patterns in mother-infant (see Van Rosmalen et al. 2015 for a review) and owner-dog dyads (e.g. Topál et al.^1998^). Interestingly, their results showed no correlation between pitch related features (mean pitch and pitch range) of dog-directed prosody and owner-dog attachment patterns (i.e. secure versus insecure, Lesch et al. 2019). These findings provide valuable contributions to the discussion. A logical step from here is to address the role of visual prosodic features, together with certain acoustic measurements, during interactions with one’s own and an unfamiliar dog.

In the present study, we aimed to investigate whether and how the presence or absence of familiarity with a dog affects acoustic and visual prosodic features of dog-directed speech. We chose to include only female dog owners as they have been shown to be more talkative and to use a more exaggerated ‘baby-talk-like’ prosodic elements with dogs (e.g. Gergely et al. 2017; Mitchell 2004; Prato-Previde et al., 2006). Being a dog owner was important in this setting, to minimize the differences in emotional responses to dogs. It has also been shown that owners perceive their dogs to be cuter than do strangers and attribute more positive personality traits and emotions to their own dogs as well as to dogs of the same breed (cf. ‘breed loyalty’, Packer et al., 2020, Thor et al., 2015). To control for this bias, we decided to observe dog-directed speech toward both the owner’s dog and an unfamiliar dog of the same breed (see Methods). The effect of familiarity on prosody has been studied only during play scenarios and the Strange situation test so far (Lesch et al. 2019; Mitchell 2004). Therefore, we wanted to examine other contexts, including an attention-getting task, an easy task-solving scenario, and a nursery rhymes situation, in line with our former comparative studies on dog-, infant- and adult-directed prosody (Gergely et al. 2017, 2023a, b).

Our research questions were as follows: (i) Does familiarity (i.e. its presence or absence) affect the acoustic and/or visual prosodic features in dog-directed speech by female speakers? (ii) Does the interaction situation (see: Methods) have an effect on the acoustic and/or visual prosodic features in dog-directed speech toward one’s own and/or an unfamiliar dog? We hypothesized that the presence or absence of familiarity would affect prosodic features in both acoustic and visual components. Based on the existing literature we predicted a higher *f0* mean (perceived as pitch, Mitchell 2004) and probably more intense facial movements and ‘happy’ expressions toward the unfamiliar dog compared to the familiar one. In this case, we would expect that the lack of familiarity elicits more intense nonverbal communication to make the human more salient to the dog. An alternative hypothesis is that bonding has no effect on dog-directed prosody. In this case we would expect no differences in the prosodic elements toward one’s own and an unfamiliar dog. Our previous studies have shown that in a similar attention-getting situation, where speakers were instructed to draw their partner’s attention to an object, the examined prosodic features were less intense compared to a task-solving situation that involved encouragement and praise (Gergely et al. 2017, 2023a, b). A situation, like telling a nursery rhyme, usually evokes exaggerated prosody with higher pitch and more intense ‘happy’ facial expressions, likely because it is linked to usually infant-directed melodic speech contexts (e.g. Koós-Hutás et al. 2024). Based on these results we expect more intense acoustic and visual prosodic elements during the *Nursery rhymes* situation followed by the *Task-solving* and finally the *Attention-getting* situation.

## Methods

### Participants

To test the potential effects of familiarity on dog-directed facial and acoustic measurements, we recruited 42 female participants (mean age ± SD: 38 ± 10.6 years) together with their pet dogs. We established the following eligibility criteria for the participants: (1) the owner must be female, and (2) the dog must belong to a breed represented by at least one other dog in the sample (in order to make pairs). Different coat and/or size variations within the same breed could be paired (see details in Table S1). We collected these voluntary participants via social media (we shared a flyer about the study and they could apply via email). Sample size (N) was based on an a-priori power analysis considering the design and statistical tests to be used (G*Power 3.1.9.7.) with ‘medium’ effect size, α = 0.05 and power (1-β) = 0.85. We used a within-subject design, in which each participant was tested while talking to their own dogs and someone else’s. To this end, we established 21 pairs of the 42 participants. Within participants with dogs of the same breed, the grouping was randomized (see Table S1). The experiments took place in the canine laboratory of the HUN-REN Research Centre for Natural Sciences, Institute of Cognitive Neuroscience and Psychology.

### Technical equipment

We used an iPhone SE 2020 smartphone for recording video and we used a microphone (Zoom F2 recorder with LMF-2 lavalier microport) to record audio. The dogs were given Platinum Lamb & Rice dry food as a treat. We also used four cameras positioned around the laboratory to record videos from different angles. The nursery rhyme was written on a piece of paper for participants to refer to.

### Procedure

Each participant had to interact with their own dog and an unfamiliar dog for about 1.5 min. Each interaction consisted of 3 situations. We have employed these 3 situations in our previous experiments (see also Gergely et al. 2017; Koós-Hutás et al. 2025) and they have proven to be reliable for differentiating acoustic and visual prosodic features among speakers. It is important to note that we named these situations in line with the instructions we gave to the owners.

1. *Attention-getting* situation (AG; 30 s): The participants were instructed to simply call (and keep) the dog’s attention to an object (the red ball or a treat mentioned above, depending on the dog’s preference).
2. *Task-solving* situation (TS; 30 s): We instructed the participants to complete an easy task with the dogs while playing a hide and seek game with the dog, using a small, red ball or a treat (depending on the dog’s preference). They hid the ball in one hand and encouraged the dog to guess which hand it was.
3. *Nursery rhymes* situation (NR; 30 sec): We asked the participants to tell the dog a well-known Hungarian nursery rhyme twice (Cini-cini muzsika; táncol a kis Zsuzsika; jobbra dől, balra dől; tücsök koma hegedül; in English: “Cini-cini music plays; little Susan dances away; leaning to the right, leaning to the left; the cricket buddy plays the fiddle”).

During all situations, the dog was held on a leash (1 m) but was not restricted in any other way. The leash was attached to a chair in which the non-speaking human was sitting. The non-speaking human was instructed to be passive, fill out a questionnaire and not to interact with the dog in any way. During the whole experiment the speaker was sitting on the floor in order to be at the same level as the dog. The speaker was asked to face the dog and not to leave the sitting position. The experimenter held the camera and followed the speaker’s face in order to maintain good video quality for the facial analysis. Both the order of conditions (someone’s own dog vs. the partner’s) and speech situations (*Task-solving*, *Attention-getting*, and *Nursery rhymes*) were counterbalanced between participants (see Table S1).

### Data analysis

The voice recordings of the speakers were analysed using Praat (Boersma and Weenink 2021), while the analysis of the obtained data of their facial movements was carried out by Noldus FaceReader software (Lewinski et al. 2014), whose automated evaluation is rooted in the Facial Action Coding System (FACS; Ekman and Friesen 1978) and is considered highly reliable (e.g., Borsos et al. 2022). Because of the automatized nature of the data processing, we did not report reliability measurements in the present study. About Praat: we used the default setting for data processing and export (i.e. 75–500 Hz range to calculate fundamental frequency mean, minimum, maximum and range, autocorrelation analysis method, automatic pitch contour drawing method). About FaceReader: the FACS coding was carried out using the built-in variables of the software (arousal, happy expression). FaceReader generates intensity values between 0 and 1 (zero to maximum intensity) on a continuous scale, which constitutes the raw data. We calculated mean intensities for each participant and each condition. These values were used in the statistical analyses.

R Studio (https://www.rstudio.com/; R version 4.3.3 using RStudio 2023.12.0 + 396) was used for statistical analysis and visualisation of the results. The acoustic (mean and range of fundamental frequency [*f0*]) and visual variables (‘arousal’ and ‘happy’ facial expressions, as defined by Noldus Face Reader) were examined by generalised linear mixed-effects modeling (glmer, lme4 package, Bates et al. 2015; Pinheiro and Bates 2000) with model comparisons via parametric bootstrapping (pbkrtest package, PBmodcomp function; Halekoh and Højsgaard 2014), since this approach provides a more robust alternative to standard χ^2^ -based Likelihood-ratio tests when dealing with mixed-effects models.

After data processing and analysis, we found that familiarity affected only one of the four variables (see Results). At the same time, the distribution and variance of the data indicated that other factors might significantly influence the results. Therefore, we decided to explore additional potential influencing factors. Previous research has suggested that traits such as baby schema (child-like facial features), small body size, and ‘cuteness’ can affect interactions with companion animals, including dogs (e.g. Borgi and Cirulli 2016). Although only two of the participating dogs were brachycephalic (i.e. having an infant-like facial appearance), the data could be balanced for body size by distinguishing between smaller (below 15 kg) and larger (above 15 kg) dogs. According to Paul and colleagues (2023), small body size contributes to ‘cuteness’ and might affect the prosodic features of dog-directed communication. Therefore, we included the dog’s size as a factor in the models.

Overall, for each dependent variable (voice: *f0 mean*, *f0 range*; face: *arousal*, *happy*) we fitted three types of models: (1) a null model containing only the random structure (speaker ID as a random intercept to control for repeated measures within speakers), (2) a full model including all fixed predictors (*condition*: own dog, unfamiliar dog; *situation*: AG, TS, NR; and *dog’s size*: below 15 kg, above 15 kg) with the same random structure, and (3) reduced models obtained from the full model by omitting one predictor at a time. Model assumptions (normality, homoscedasticity, linearity, and influential points) were checked using the check_model() function (*performance* package, Lüdecke et al. 2021). Variance inflation factors (VIFs) were all below 2, indicating no multicollinearity. Model comparisons were performed with parametric bootstrap likelihood ratio tests (*PBmodcomp*,* pbkrtest*) consisting of 1000 simulations. Model fit was evaluated based on AIC values - lower AIC indicating a better fit. Each reduced and null model was compared to the full model (see Table 1). A significant PBtest (*p* < 0.05) was interpreted as evidence for a significantly worse model fit for the reduced model than for the full model, indicating that the removed predictor significantly contributed to model performance. Based on the preliminary analysis, fixed variables’ 3-way (*condition* × *situation* × *dog’s size*) and 2-way interactions (*condition* × *situation*, *situation* × *dog’s size*, *dog’s size* × *condition*) did not significantly affect the measured variables. Therefore, the final analysis provides evidence only of the main effects. Because parametric bootstrapping is relatively robust to deviations from a normal distribution, and the diagnostic checks of model residuals for mildly skewed variable (*happy*,* f0 range*) did not indicate substantial violations of model assumptions - i.e. the central part of the distribution remained approximately normal, we used Gaussian models with a log link function for all analyses. In case of significant effect, post-hoc pairwise comparisons were performed using the *emmeans* package (Lenth, 2023), with Tukey-adjusted p-values. Effect sizes (Cohen’s d) were calculated for all pairwise contrasts using the residual standard deviation from the model. (We have included these in the tables in the Results section.)

**Table 1:** List of models tested throughout the analysis with Akaike information criterion (AIC)

Dependent variable	model	AIC	ΔAIC	Note
F0 mean	null	2516.7	84.6	
	reduced (condition + situation)	2437.6	5.5	best fitting
	full	2432.1	0.0	
F0 range	null	2757.1	7.4	
	reduced (situation + dog’s size)	2748.7	0.0	best fitting
	full	2749.4	0.7	
Arousal	null	-635.1	86.3	
	reduced (situation)	-721.4	0.0	best fitting
	full	-719.2	2.2	
Happy	null	-430.6	37.2	
	reduced (situation + dog’s size)	-457.8	0.0	best fitting
	full	-457.2	0.6	

A final note: the amount of speech can be an important and a potential influencing factor. Thus we annotated 28% of the recordings, namely in 12 speakers both in the participant’s own and unfamiliar dog conditions in the two free speech situations (*Attention-getting* and *Task-solving*), as in the fixed speech (i.e. *Nursery rhymes*) situation the same words were said the same number of times by all speakers. Next we counted the spoken words and calculated a word/second ratio for each video in order to make such an analysis. We found no significant effect of the condition (own vs. unfamiliar dog) on the speech amount of the speakers (i.e. word/sec. ratio; GLMM F_1,46_=0.33, *p* = 0.57).

## Results

### Acoustic measurements

**Fig. 1:**
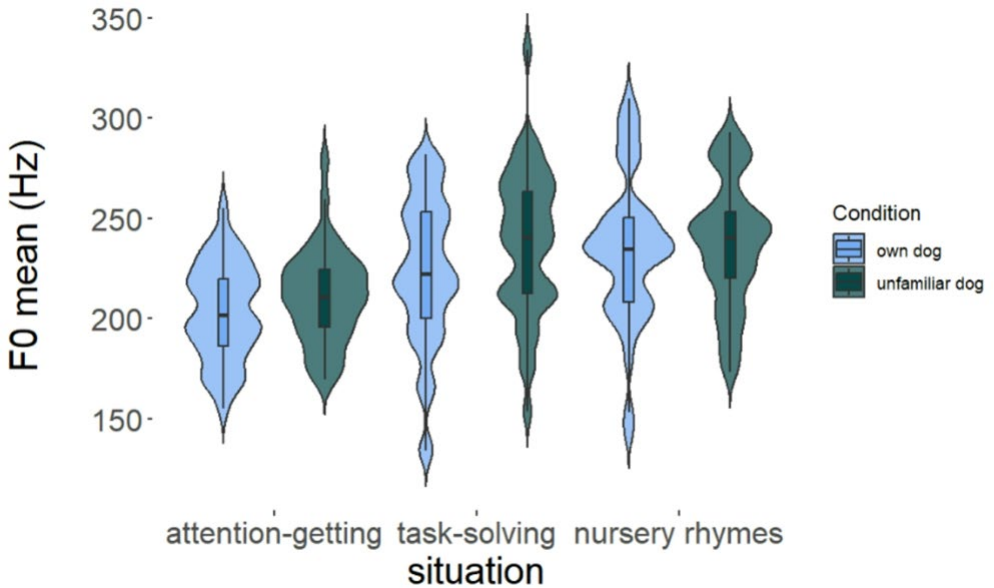
The mean level of fundamental frequency (f0, in Hz) during situations and according to conditions. [Within the boxplots, the horizontal line represents the median, the box shows the quartiles, the whiskers represent the range, and the violin plots depict the density of the data. Conditions: light blue – own dog; dark green – unfamiliar dog.]

Comparing the full model (including condition, situation and dog’s size) for mean fundamental frequency (*f0 mean)* to a reduced one showed that the model fit significantly worsened with removing condition (*PBtest*: χ^2^_1_ = 10.778, *p* < 0.01) or situation (*PBtest*: χ^2^_2_ = 65.978, *p* < 0.001). The pairwise comparison unveiled an overall higher mean fundamental frequency toward unfamiliar, than toward own dogs, irrespective of the given situation (*p* = 0.001, see Fig. 1; Table 2, for detailed statistics see Table 3). In line with our a-priori prediction and regardless of the presence or absence of familiarity, speakers used the lowest *f0 mean* during *Attention-getting* scenarios (*Attention-getting* vs. *Task-solving*, *Attention-getting* vs. *Nursery rhymes* both *p* < 0.001, Fig. 1). At the same time, speakers used a similar *f0 mean* when telling a nursery rhyme and completing the *Task-solving* situation, regardless of whether the dog was their own or unfamiliar (*p* = 0.292, see Fig. 1; Table 2, for detailed statistics see Table 3).

**Table 2:** Summary of the results. *f0* = fundamental frequency, NR = *Nursery rhymes* situation, TS = *Task-solving* situation, AG = *Attention-getting* situation, small = dogs below 15 kg, big = dogs above 15 kg, Ns.=non-significant

	Acoustic measurements			Visual measurements	
	f0 mean	f0 range	Arousal		Happy
Condition (Familiarity)	Unfamiliar dog > own dog	Ns.	Ns.		Ns.
Situation	(NR = TS) > AG	(TS > NR) = AG	NR> (TS = AG)		NR> (TS = AG)
Dog’s size	Ns.	Small > big	Ns.		Small > big

**Table 3:** Pairwise comparisons of conditions and situations on the *mean fundamental frequency* (condition: OWN – own dog, UNF – unfamiliar dog; situation: AG – attention-getting, TS – task solving, NR – nursery rhyme)

condition	OWN vs. UNF	β ± SE = -9.16 ± 2.77, t = -3.304, *p* = 0.001	Cohen’s d= -0.411, 95% CI (-0.659, -0.164)
situation	**AG vs. TS**	β ± SE = -22.94 ± 3.40, t = -6.741, ***p*** **< 0.001**	Cohen’s d= -1.028, 95% CI (-1.342, -0.714)
	**AG vs. NR**	β ± SE = -27.99 ± 3.42, t= -8.178, ***p*** **< 0.001**	Cohen’s d= -1.254, 95% CI (-1.576, -0.932)
	**TS vs. NR**	β ± SE = -5.05 ± 3.36, t= -1.502, *p* = 0.292	Cohen’s d= -0.226, 95% CI (-0.524, 0.071)

**Fig. 2:**
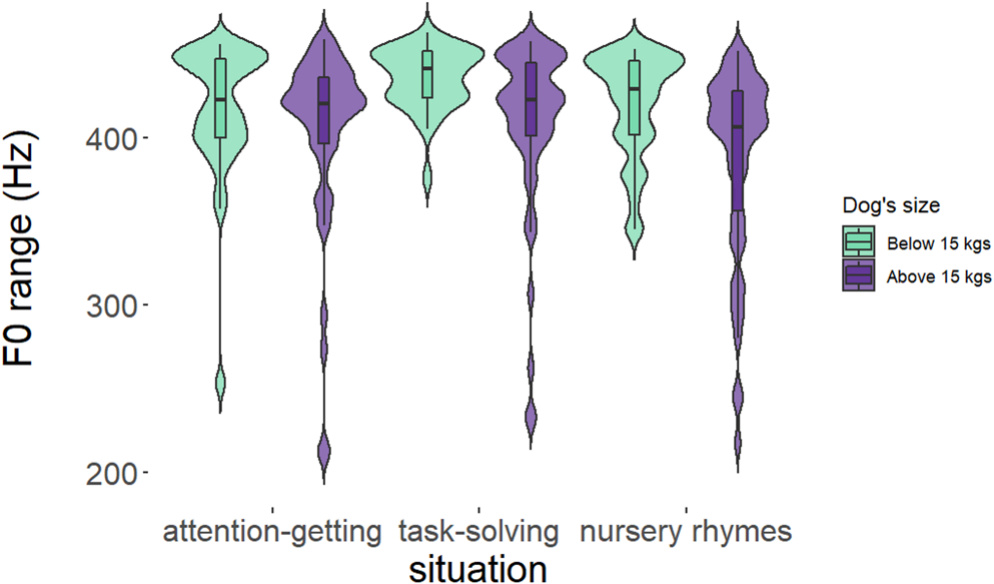
The range of fundamental frequency (f0, in Hz) during situations and according to the dog’s size. [Within the boxplots, the horizontal line represents the median, the box shows the quartiles, the whiskers represent the range, and the violin plots depict the density of the data. Dog’s size: light green – below 15 kg; purple – above 15 kg.]

In the case of the *f0 range*, there was significant improvement in model fit in the presence of the situation (*PBtest*: χ^2^_2_ = 11.09, *p* = 0.006). Moreover, the model comparison revealed the dog’s size to be a significant predictor as well (*PBtest*: χ^2^_1_ = 6.713, *p* = 0.015). Post-hoc pairwise comparison showed that speakers used a significantly wider pitch range in *Task-solving* than in *Nursery rhymes* situation (*p* = 0.003, see Fig. 2; Table 2, for detailed statistics see Table 4). However, *Attention-getting* did not differ significantly from either *Nursery rhymes* (*p* = 0.246) or *Task-solving* (*p* = 0.215). Nevertheless, when comparing the two types of dog sizes, speakers used a wider pitch range while interacting with smaller dogs, regardless of the situation (*p* = 0.012, see Fig. 2; Table 2, for detailed statistics see Table 4).

**Table 4:** Pairwise comparisons of situations and dog size on the range of fundamental frequency (situation: AG—attention-getting, TS—task solving, NR—nursery rhyme)

dog size	< 15 kg vs. >15 kg	β ± SE = 25.4 ± 9.66, t = 2.636, *p* = 0.012	Cohen’s d = 0.002, 95% CI (0.0006, 0.0019)
situation	**AG vs. TS**	β ± SE = -10.08 ± 6.00, t = -1.680, *p* = 0.215	Cohen’s d= -0.0005, 95% CI (-0.0012, 0.0001)
	**AG vs. NR**	β ± SE = -9.69 ± 6.03, t = 1.605, *p* = 0.246	Cohen’s d = 0.0004, 95% CI (-0.0002, 0.0012)
	**TS vs. NR**	β ± SE = 19.76 ± 5.91, t = 3.347, ***p*** **= 0.003**	Cohen’s d = 0.001, 95% CI (0.0003, 0.0017)

### Visual measurements

**Fig. 3:**
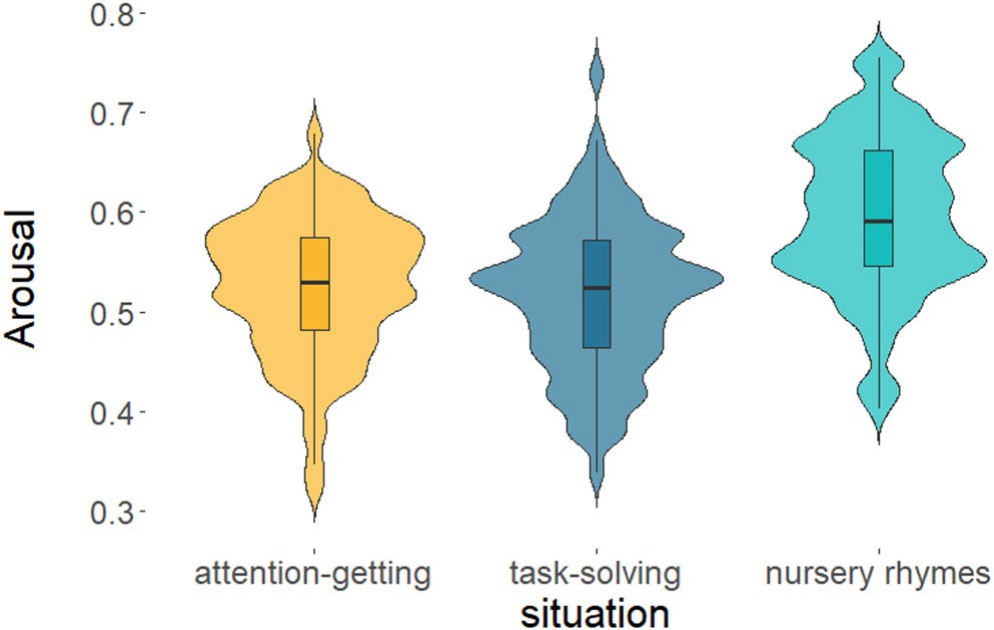
The mean intensity level of Arousal during situations across conditions and the dog’s size. [Within the boxplots, the horizontal line represents the median, the box shows the quartiles, the whiskers represent the range, and the violin plots depict the density of the data. Situations: yellow – Attention-getting; dark blue – Task-solving; turquoise – Nursery rhymes.]

For the indicator of any involvement of facial muscles– i.e. *Arousal*, the parametric bootstrap-based model comparison showed only a significant situation main effect (*PBtest*: χ^2^_2_ = 82.882, *p* < 0.001), but it is worth noting the effect of dog’s size as a trend; nonetheless, it was eliminated from the final model (*PBtest*: χ^2^_1_ = 3.064, *p* = 0.093). Post-hoc comparison between situations showed more intense facial movements during *Nursery rhymes* than during *Attention-getting* and *Task-solving* (both *p* < 0.001, see Table 2; Fig. 3, for detailed statistics see Table 5). The *arousal* level of the speakers’ faces did not differ between *Attention-getting* and *Task-solving* situations (*p* = 0.611).

**Table 5:** Pairwise comparisons of situations on the arousal of the face with effect sizes (situation: AG—attention-getting, TS—task solving, NR—nursery rhyme)

situation	AG vs. TS	β ± SE = 0.0082 ± 0.0086, t = 0.948, *p* = 0.611	Cohen’s d = 0.251, 95% CI (-0.27, 0.77)
	**AG vs. NR**	β ± SE = -0.0692 ± 0.0087, t = -7.995, ***p*** **< 0.001**	Cohen’s d= -2.103, 95% CI (-2.63, -1.58)
	**TS vs. NR**	β ± SE = -0.0774 ± 0.0085, t = -9.100, ***p*** **< 0.001**	Cohen’s d= -2.354, 95% CI (-2.88, -1.83)

**Fig. 4:**
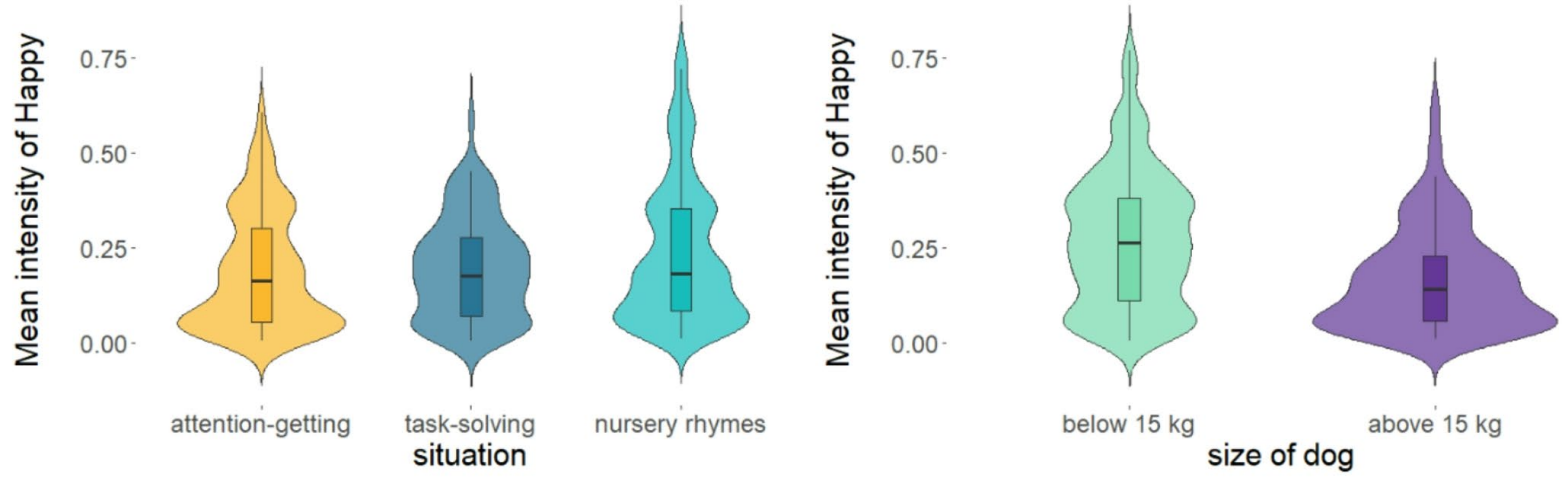
The mean intensity level of ‘happy’ facial expressions during situations (**a**) and according to the dog’s size (**b**). [Within the boxplots, the horizontal line represents the median, the box shows the quartiles, the whiskers represent the range, and the violin plots depict the density of the data. Situations: yellow – Attention-getting; dark blue – Task-solving; turquoise – Nursery rhymes. Dog’s size: light green – below 15 kg; purple – above 15 kg.]

The parametric bootstrap test for ‘happy’ facial expressions indicated a significant main effect of the situation (*PBtest*: χ^2^_2_ = 16.778, *p* < 0.001) and the dog’s size (*PBtest*: χ^2^_1_ = 7.0299, *p* = 0.013). Regarding the pairwise comparison, the post-hoc analysis revealed that the general mean intensity of ‘happy’ facial expressions was significantly higher during *Nursery rhymes* than in *Attention-getting* and in *Task-solving* situations (both *p* < 0.01, see Table 2; Fig. 4a, for detailed statistics, see Table 6). Meanwhile, between the latter two, there was no significant difference (*p* = 0.541). Furthermore, irrespective of the situation, more intense ‘happy’ facial expressions were exhibited by the speakers towards dogs weighing less than 15 kg (*p* = 0.01, see Table 2; Fig. 4b, for detailed statistics, see Table 6).

**Table 6:** Pairwise comparisons of situations and dog size on the happy facial expressions with effect sizes (situation: AG—attention-getting, TS—task solving, NR—nursery rhyme)

dog size	< 15 kg vs. >15 kg	β ± SE = 0.107 ± 0.0398, t = 2.699, *p* = 0.01	Cohen’s d = 2.61, 95% CI (-2.54,7.75)
situation	**AG vs. TS**	β ± SE = -0.0152 ± 0.0143, t = -1.059, *p* = 0.541	Cohen’s d= -0.067, 95% CI [-1.16, 1.03]
	**AG vs. NR**	β ± SE = -0.0574 ± 0.0144, t = -3.984, ***p*** **< 0.001**	Cohen’s d= -2.539, 95% CI (-3.55, -1.52)
	**TS vs. NR**	β ± SE = -0.0422 ± 0.0142, t = -2.984, ***p*** **= 0.009**	Cohen’s d= -2.472, 95% CI (-3.47, -1.47)

## Discussion

The aim of the present experiment was to investigate whether, and how, familiarity and/or the given situation have an effect on acoustic and visual prosodic features of female speakers toward dogs. To achieve this, speakers interacted with both their own dog and an unfamiliar dog of the same breed in three different situations. During the analysis, we expanded the scope to include the size of the dogs as a potential influencing factor on prosodic elements.

Let us look at what we can conclude regarding the effects of familiarity. Interestingly, it only affected the mean pitch (i.e. fundamental frequency, *f0*) of the speakers, which aligns with our hypothesis and a previous study. Specifically, the mean pitch was higher toward unfamiliar dogs compared to participants’ own dogs (Mitchell 2004). The most likely explanation for this phenomenon is as follows: According to the existing literature, *f0* mean appears to be a universal communicative tool in inter-species communication, as humans tend to use elevated pitch when talking to dogs, cats and parrots (e.g. Burnham et al. 2002; Gergely et al. 2017; Xu et al. 2013). This might be linked to ancient evolutionary processes, as high-pitched clear sounds serve as attention-getting devices used by many species during friendly encounters as well as in alarm calls (e.g. Hauser 1993; Mitchell et al. 2025; Morton 1977). Higher frequencies in vocalisation is also considered to be a sign of higher arousal across species (Briefer 2020). It seems logical to pay more attention to an individual that is more aroused, because they are more likely to possess important information about the environment, pose more threat etc., hence the attention-provoking nature of a higher *f0*. We suggest that, in heterospecific communication, it makes sense to rely on universal signals that are more likely to be understood and responded to, rather than signals that may work well with conspecifics but are less effective with other species. Our results suggest that, in the absence of familiarity, speakers rely more on this universal acoustic tool (i.e. heightened *f0* mean) to engage an unfamiliar canine partner. Elevated pitch, therefore, may be employed to communicate friendly intentions toward an unfamiliar partner, at least for female speakers. Showing friendly intentions may be important, because unfamiliar partners have no previous experience with us and might as well find us threatening. Another possible explanation for the differences between own and unfamiliar dogs is that the owner and the unfamiliar dog do not share a common set of relation-specific signals (verbal or non-verbal) that could facilitate the communication, therefore, they have to rely on more general prosodic cues.

At the same time, and contrary to our predictions, familiarity had no effect on visual prosodic measurements. One possible explanation is that visual prosody may primarily involve signals reserved for conspecifics. In line with this assumption, previous comparative studies have shown that speakers tend to use more frequent and intense positive facial expressions when talking to other humans, but less so when talking to dogs (Gergely et al. 2023a, b; Koós-Hutás et al. 2024). Furthermore, it has been proposed that several facial signals commonly associated with happy and surprised facial expressions in humans (i.e. showing teeth and gums, eyebrow raise, wide opened eyes) convey aggressive or agonistic messages in canines (Koós-Hutás et al. 2024). Therefore, it is plausible to assume that speakers tend to reduce these facial signals when interacting with dogs regardless of their relationship with the animal (i.e. whether they are familiar with them or not). Finally, it is worth considering that, when interacting with dogs, the human face is often not in the focus of the dog’s visual field (as opposed to when communicating with another human), therefore, the human partner (perhaps unconsciously) capitalizes on their voice instead of their face.

Apart from the effects of familiarity, the second question we addressed was whether the nature of the situation (i.e. *Attention-getting*, *Task-solving* and *Nursery rhymes*) has an effect on the characteristics of the acoustic and/or visual prosodic features. In line with our hypotheses, we found that the situation had a significant main effect on all examined prosodic variables. It should be noted that the *Task-solving* situation might also have some attention-provoking nature, as the dog has to attend to the task at hand. Nevertheless, a task is likely to grab the dog’s attention more naturally, so we assume less direct attention-getting was needed in this situation. Now we will elaborate on the most important findings in this regard.

Firstly, mean *f0* was significantly lower in the *Attention-getting* situation compared to the *Task-solving* and *Nursery rhymes* situations. This effect aligns with previous studies and further supports the notion that in situations in which speakers are instructed to draw the partner’s attention to an object, a less intense prosody is observed. None of the examined prosodic variables were more prominent in this situation. This result might suggest that it is the lexical content and/or the motions and gestures employed by the speakers that play a key role in directing the partner’s attention toward the objects (and not the prosodic features per se). The fact that the *f0* mean is specifically lower in such situations might have to do with a high *f0* mean’s attention-provoking nature (i.e. it would direct the partner’s attention to the speaker instead of the object) (e.g. Jeannin et al. 2017; Koós-Hutás et al. 2025), which, in turn, might stem from the higher arousal attributed to a higher *f0* (Briefer 2020). Further studies could help clarify the role of lexical cues and bodily motions in such situations to better understand how speakers direct the canine partner’s attention from themselves to an object.

Secondly, in the *Nursery rhymes* situation, we expected more intense acoustic and / or visual prosodic measurements. Of these two, the visual features were, indeed, more intense; both the level of arousal and the frequency of the ‘happy’ expression were significantly higher during rhyming. One plausible explanation lies in the infant-related nature of the task itself. Namely, the infant-directedness of a given situation has the potential to evoke ‘infant-related’ prosody, even toward dogs (Koós-Hutás et al. 2024, 2025). Another influencing factor could have been the rhythmical / musical nature of the utterances subjects had to produce. There are data that rhythm and, in a wider sense, musical entrainment has the capability to alter bodily functions and invoke emotions (Nozaradan 2014; Trost and Vuilleumier 2013), which, in turn, might also affect visual prosody. Finally, the nursery rhyme might evoke positive memories in the speakers.

Actually there is only one result that does not align with our hypotheses and prior predictions: the *f0* range was wider in the *Task-solving* than in the *Nursery rhymes* situation. We assume that in *Task-solving*, emotional utterances are needed to engage the partner and encourage success (i.e. through praise, encouragement or signaling when they did not succeed). It appears that wider *f0* range serves these functions (i.e. communicating emotions, influencing behavior, and providing reward), while during *Nursery rhymes*, speakers employ a high but less variable pitch. It seems as if the *f0* range specifically serves to communicate emotional cues and influence the partner’s behavior. Let us not forget that the *Nursery rhymes* situation was the only one that involved reading, so it is safe to say that it was the least spontaneous situation. Also it could easily have been the most unusual of all the situations. These subjective factors might also have played an influential role in modifying the prosodic features observed.

Finally, as a post-hoc decision, we included the dogs’ body size as a potential influencing factor. It turned out that two of the examined metrics – intensity of the ‘happy’ facial expression and the *f0* range – were affected by the size of the dog. Both of them were significantly greater towards smaller dogs (i.e. those under 15 kg). According to Paul and colleagues (2023), a smaller size contributes to ‘cuteness’, and it seems reasonable to assume that this drives the above-mentioned changes. One may also assume that small-sized dogs bear more resemblance to human infants, triggering signals typically associated with infant-directed behaviours. Alternatively, it is also possible that our small-dog sample contained more dogs with explicit paedomorphic features (e.g. raccoon-eyes, small nose, big eyes, big forehead, etc., e.g. Dale et al. 2016; Forman et al. 2023; Paul et al. 2023) compared to the larger dogs and that these features, rather than body size alone, may have contributed to the more exaggerated prosodic features. Therefore, future research should more systematically examine the effect of dogs’ body size, paedomorphic features and perceived ‘cuteness’ on dog-directed communication.

At this point, it might be worth mentioning that, compared with adult-infant communication, the prosodic features in human-dog interactions seem to follow different patterns. They are often compared to one another, due to obvious similarities (e.g. Mitchell 2001), yet one crucial difference is that with an unfamiliar canine partner (see Mitchell 2004 or the present results), the intensity of prosodic elements increases, whereas it decreases with unfamiliar adult humans (Gergely et al. 2023a, b; Koós-Hutás et al. 2025). (There is currently no evidence comparing familiar and unfamiliar infants.) The most plausible reason is that speakers wish to appear more friendly toward an unfamiliar dog, who has no previous experience with them, to reassure the dog about their friendly intentions (Mitchell 2004). We think that humans might not need this kind of reassurance, as we normally do not suppose that an unfamiliar (but otherwise decent and ordinary) person would do us any harm.

The key takeaway is that humans seem to differentiate between canine partners in a given situation based on factors like familiarity and ‘cuteness’ and align their communication style to the partner’s and the relationship’s characteristics. This hints at the complexity of human-dog relationships and invites us to think about and explore its depths and nuances even further.

## Supplementary Information

Below is the link to the electronic supplementary material.

Supplementary Material 1

Supplementary Material 2

Supplementary Material 3

## Data Availability

No datasets were generated or analysed during the current study.
