# Supplementary material for: The effect of familiarity and dog’s body size on female owners’ dog-directed communication
Source: Anim Cogn. 2026 Jan 8;29(1):16. doi: 10.1007/s10071-025-02041-1 (PMC12823717; doi:10.1007/s10071-025-02041-1)
Supplement: Supplementary file 2 — Supplementary Material 2 [file 10071_2025_2041_MOESM2_ESM.pdf]

SUPPLEMENTARY MATERIAL

The Effect of Bonding and Dog's Body Size on female owners' Dog-Directed Communication

Lőrinc A. Filep, Édua Koós-Hutás, Fanni Hollay, József Topál & Anna Gergely

Table S1. List of participants and experimental orders of conditions and situations. ID= identification number, O=own dog condition, U=unfamiliar dog condition, A=attention getting situation, T=task solving situation, N=nursery rhyme situation

Group ID	Speaker ID	Dog breed	Dog sex	Dog age (years)	Dog height (at withers, cm)	Dog weight (kg)	Order of conditions	Order of situations
1	1	Belgian Malinois	Intact male	3	61		O, U	N, A, T<br>T, A, N
	2	Belgian Malinois	Intact male	2	60		U, O	A, N, T<br>T, A, N
2	3	Mudi	Intact male	4	44		O,U	N, T,A<br>N,A,T
	4	Mudi	Intact female	3	43		U,O	A,N,T<br>T,N,A
3	5	Bullterrier	Spayed male	10	52		U,O	A,N,T<br>T,A,N

	6	Bullterrier (Miniature)	Intact male	1	36		O,U	T,N,A<br>N,T,A
4	7	Golden retriever	Spayed male	8	59		O,U	A,N,T<br>N,A,T
	8	Golden retriever	Intact female	3	55		U,O	T,N,A<br>N,A,T
5	9	Border Collie	Intact male	2	54		O,U	N,T,A<br>A,N,T
	10	Border Collie	Intact male	3	54		U,O	T,N,A<br>T,A,N
6	11	Border Collie	Spayed female	9	48		U,O	A,N,T<br>T,A,N
	12	Border Collie	Spayed female	2	47		O,U	A,T,N<br>N,A,T
7	13	Poodle (Miniature)	Spayed female	1	33		O,U	T,A,N<br>T,N,A
	14	Poodle (Medium)	Intact female	2	38		U,O	N,T,A<br>A,N,T
8	15	Portuguese waterdog	Spayed female	4	45	17	O,U	N,A,T<br>T,A,N

	16	Portuguese waterdog	Intact male	2	56	20	U,O	A,N,T<br>N,T,A
9	17	Samoyed	Intact female	3	55	26	U,O	T,A,N<br>T,N,A
	18	Samoyed	Spayed female	4	55	23	O,U	A,N,T<br>N,T,A
10	19	Bichon havanese	Intact female	2	23	5	U,O	N,A,T<br>N,A,T
	20	Bichon havanese	Spayed male	2	24	8	O,U	T,A,N<br>A,T,N
11	21	Miniature schnauzer	Spayed female	5	32	7,5	O,U	T,N,A<br>T,A,N
	22	Miniature schnauzer	Intact male	2	33	7	U,O	A,T,N<br>A,N,T
12	23	Hungarian vizsla	Spayed male	7	57	25	O,U	A,N,T<br>T,N,A
	24	Hungarian vizsla (wired-haired)	Spayed female	3	58	23	U,O	T,A,N<br>T,N,A
13	25	Dachshund	Intact male	1	27	8	U,O	N,A,T

		(Wire Haired)						N,A,T
	26	Dachshund	Spayed male	2	20	9	O,U	T,N,A<br>A,N,T
14	27	Australian Shepherd	Spayed female	2	53	20	O,U	T,N,A<br>N,A,T
	28	Australian Shepherd	Spayed female	4	46	20	U,O	A,T,N<br>A,N,T
15	29	Australian Shepherd	Intact male	2	51	25	U,O	N,T,A<br>A,T,N
	30	Australian Shepherd	Spayed male	5	51	26	O,U	A,N,T<br>T,N,A
16	31	Hungarian greyhound	Spayed female	9	68	30	O,U	T,A,N<br>T,A,N
	32	Hungarian greyhound	Intact male	2	70	29	U,O	N,A,T<br>A,N,T
17	32	Siberian Husky	Spayed female	2	57	19	O,U	A,N,T<br>T,A,N
	34	Siberian Husky	Spayed female	7	60	21	U,O	N,T,A<br>T,N,A

18	35	Pug	Intact male	6	32	8	O,U	A,N,T<br>T,A,N
	36	Pug	Intact male	2	34	10	U,O	T,N,A<br>N,T,A
19	37	Jack Russel Terrier	Spayed male	3	32	10	U,O	A,N,T<br>T,N,A
	38	Jack Russel Terrier	Spayed male	2	28	7	O,U	T,N,A<br>N,T,A
20	39	Australian kelpie	Spayed male	3	53	17	O,U	N,A,T<br>T,N,A
	40	Australian kelpie	Spayed female	3	52	17	U,O	T,N,A<br>A,N,T
21	41	Miniature Pinscher	Spayed male	2	32	5	O,U	N,A,T<br>T,N,A
	42	Miniature Pinscher	Spayed male	1	31	6	U,O	T,A,N<br>N,A,T
