# Supplementary material for: The effect of familiarity and dog’s body size on female owners’ dog-directed communication
Source: Anim Cogn. 2026 Jan 8;29(1):16. doi: 10.1007/s10071-025-02041-1 (PMC12823717; doi:10.1007/s10071-025-02041-1)
Supplement: Supplementary file 3 — Supplementary Material 3 [file 10071_2025_2041_MOESM3_ESM.docx]

**SUPPLEMENTARY MATERIAL**

The Effect of Bonding and Dog’s Body Size on female owners’ Dog-Directed Communication

Lőrinc A. Filep, Édua Koós-Hutás, Fanni Hollay, József Topál & Anna Gergely

**Table S1.** List of participants and experimental orders of conditions and situations. ID= identification number, O=own dog condition, U=unfamiliar dog condition, A=attention getting situation, T=task solving situation, N=nursery rhyme situation

| Group ID | Speaker ID | Dog breed | Dog sex | Dog age (years) | Dog height (at withers, cm) | Dog weight (kg) | Order of conditions | Order of situations |
| --- | --- | --- | --- | --- | --- | --- | --- | --- |
| 1 | 1 | Belgian Malinois | Intact male | 3 | 61 |  | O, U | N, A, T  T, A, N |
|  | 2 | Belgian Malinois | Intact male | 2 | 60 |  | U, O | A, N, T  T, A, N |
| 2 | 3 | Mudi | Intact male | 4 | 44 |  | O,U | N, T,A  N,A,T |
|  | 4 | Mudi | Intact female | 3 | 43 |  | U,O | A,N,T  T,N,A |
| 3 | 5 | Bullterrier | Spayed male | 10 | 52 |  | U,O | A,N,T  T,A,N |
|  | 6 | Bullterrier  (Miniature) | Intact male | 1 | 36 |  | O,U | T,N,A  N,T,A |
| 4 | 7 | Golden retriever | Spayed male | 8 | 59 |  | O,U | A,N,T  N,A,T |
|  | 8 | Golden retriever | Intact female | 3 | 55 |  | U,O | T,N,A  N,A,T |
| 5 | 9 | Border Collie | Intact male | 2 | 54 |  | O,U | N,T,A  A,N,T |
|  | 10 | Border Collie | Intact male | 3 | 54 |  | U,O | T,N,A  T,A,N |
| 6 | 11 | Border Collie | Spayed female | 9 | 48 |  | U,O | A,N,T  T,A,N |
|  | 12 | Border Collie | Spayed female | 2 | 47 |  | O,U | A,T,N  N,A,T |
| 7 | 13 | Poodle  (Miniature) | Spayed female | 1 | 33 |  | O,U | T,A,N  T,N,A |
|  | 14 | Poodle  (Medium) | Intact female | 2 | 38 |  | U,O | N,T,A  A,N,T |
| 8 | 15 | Portuguese waterdog | Spayed female | 4 | 45 | 17 | O,U | N,A,T  T,A,N |
|  | 16 | Portuguese waterdog | Intact male | 2 | 56 | 20 | U,O | A,N,T  N,T,A |
| 9 | 17 | Samoyed | Intact female | 3 | 55 | 26 | U,O | T,A,N  T,N,A |
|  | 18 | Samoyed | Spayed female | 4 | 55 | 23 | O,U | A,N,T  N,T,A |
| 10 | 19 | Bichon havanese | Intact female | 2 | 23 | 5 | U,O | N,A,T  N,A,T |
|  | 20 | Bichon havanese | Spayed male | 2 | 24 | 8 | O,U | T,A,N  A,T,N |
| 11 | 21 | Miniature schnauzer | Spayed female | 5 | 32 | 7,5 | O,U | T,N,A  T,A,N |
|  | 22 | Miniature schnauzer | Intact male | 2 | 33 | 7 | U,O | A,T,N  A,N,T |
| 12 | 23 | Hungarian vizsla | Spayed male | 7 | 57 | 25 | O,U | A,N,T  T,N,A |
|  | 24 | Hungarian vizsla  (wired-haired) | Spayed female | 3 | 58 | 23 | U,O | T,A,N  T,N,A |
| 13 | 25 | Dachshund  (Wire Haired) | Intact male | 1 | 27 | 8 | U,O | N,A,T  N,A,T |
|  | 26 | Dachshund | Spayed male | 2 | 20 | 9 | O,U | T,N,A  A,N,T |
| 14 | 27 | Australian Shepherd | Spayed female | 2 | 53 | 20 | O,U | T,N,A  N,A,T |
|  | 28 | Australian Shepherd | Spayed female | 4 | 46 | 20 | U,O | A,T,N  A,N,T |
| 15 | 29 | Australian Shepherd | Intact male | 2 | 51 | 25 | U,O | N,T,A  A,T,N |
|  | 30 | Australian Shepherd | Spayed male | 5 | 51 | 26 | O,U | A,N,T  T,N,A |
| 16 | 31 | Hungarian greyhound | Spayed female | 9 | 68 | 30 | O,U | T,A,N  T,A,N |
|  | 32 | Hungarian greyhound | Intact male | 2 | 70 | 29 | U,O | N,A,T  A,N,T |
| 17 | 32 | Siberian Husky | Spayed female | 2 | 57 | 19 | O,U | A,N,T  T,A,N |
|  | 34 | Siberian Husky | Spayed female | 7 | 60 | 21 | U,O | N,T,A  T,N,A |
| 18 | 35 | Pug | Intact male | 6 | 32 | 8 | O,U | A,N,T  T,A,N |
|  | 36 | Pug | Intact male | 2 | 34 | 10 | U,O | T,N,A  N,T,A |
| 19 | 37 | Jack Russel Terrier | Spayed male | 3 | 32 | 10 | U,O | A,N,T  T,N,A |
|  | 38 | Jack Russel Terrier | Spayed male | 2 | 28 | 7 | O,U | T,N,A  N,T,A |
| 20 | 39 | Australian kelpie | Spayed male | 3 | 53 | 17 | O,U | N,A,T  T,N,A |
|  | 40 | Australian kelpie | Spayed female | 3 | 52 | 17 | U,O | T,N,A  A,N,T |
| 21 | 41 | Miniature Pinscher | Spayed male | 2 | 32 | 5 | O,U | N,A,T  T,N,A |
|  | 42 | Miniature Pinscher | Spayed male | 1 | 31 | 6 | U,O | T,A,N  N,A,T |
